# Intelligent air defense task assignment based on hierarchical reinforcement learning

**DOI:** 10.3389/fnbot.2022.1072887

**Published:** 2022-12-01

**Authors:** Jia-yi Liu, Gang Wang, Xiang-ke Guo, Si-yuan Wang, Qiang Fu

**Affiliations:** Air and Missile Defense College, Air Force Engineering University, Xi’an, China

**Keywords:** air defense task assignment, hierarchical reinforcement learning, model predictive control, proximal policy optimization, agent

## Abstract

Modern air defense battlefield situations are complex and varied, requiring high-speed computing capabilities and real-time situational processing for task assignment. Current methods struggle to balance the quality and speed of assignment strategies. This paper proposes a hierarchical reinforcement learning architecture for ground-to-air confrontation (HRL-GC) and an algorithm combining model predictive control with proximal policy optimization (MPC-PPO), which effectively combines the advantages of centralized and distributed approaches. To improve training efficiency while ensuring the quality of the final decision. In a large-scale area air defense scenario, this paper validates the effectiveness and superiority of the HRL-GC architecture and MPC-PPO algorithm, proving that the method can meet the needs of large-scale air defense task assignment in terms of quality and speed.

## Introduction

Modern air defense operations are becoming more complex with the rapid development of long-range, elemental, and intelligent processes. The rational planning of interception plans for incoming air targets to maximize operational effectiveness has become a massive challenge for the defenders in modern air defense operations (Yang et al., 2019). Task assignment changes the weapon target assignment (WTA) fire unit-target model to a task-target assignment model. This improves the ability to coordinate the various components, and the assignment scheme is more flexible, providing fundamental assurance of maximum operational effectiveness (Wang et al., 2019). With the continuous adoption of new technologies on both sides of the battlefield, the combat process is becoming increasingly complex, involving many elements. The battlefield environment and the adversary’s strategy are rapidly changing and challenging to quantify. Relying on human judgment and decision-making can no longer adapt to the requirements of fast-paced and high-intensity confrontation, and depending on traditional analytical model processing cannot adapt to the needs of complex and changing scenarios. Reinforcement learning (RL) does not require an accurate mathematical model of the environment and the task and is less dependent on external guidance information. Therefore, some scholars have investigated the task assignment problem through intelligent methods such as single-agent reinforcement learning, multi-agent reinforcement learning (MARL), and deep reinforcement learning (DRL). Zhang et al. (2020) proposed an Imitation augmented deep reinforcement learning (IADRL) model to enable unmanned aerial vehicles (UAVs) and unmanned ground vehicles (UGVs) to form a complementary and cooperative alliance to accomplish tasks that they cannot do alone. Wu et al. (2022) proposed a dynamic multi-UAV task assignment algorithm based on reinforcement learning and a deep neural network, which effectively solves the problem of poor mission execution quality in complex dynamic environments. Zhao et al. (2019) proposed a Q-learning-based fast task assignment (FTA) algorithm for solving the task assignment problem of heterogeneous UAVs.

In modern air defense operations, the threat to the defense can be either a large-scale air attack or a small-scale contingency, so mission assignment methods must balance effectiveness and dynamism. A centralized assignment solution is not fast enough, while a fully distributed assignment method does not respond effectively to unexpected events (Lee et al., 2012). The one-general agent with multiple narrow agents (OGMN) architecture proposed in the literature (Liu J. Y. et al., 2022), which divides agents into general and narrow agents, improves the computational speed and coordination ability. However, the narrow agent in the OGMN is entirely rule-driven. It lacks a certain degree of autonomy, which cannot fully adapt to the complex and changing battlefield environment. Therefore, this paper proposes the hierarchical reinforcement learning architecture for ground-to-air confrontation (HRL-GC) architecture based on the OGMN architecture, which layers the agents into scheduling and execution. The scheduling agent is responsible for assigning targets to the execution agent, which makes the final decision based on its state. Data drive both types of agents. Considering the inefficiency of the initial phase of agents training, this paper proposes a model-based model predictive control with proximal policy optimization (MPC-PPO) algorithm to train the execution agent to reduce inefficient exploration. Finally, the HRL-GC is compared with two other architectures in a large-scale air defense scenario, and the effectiveness of the MPC-PPO algorithm is verified. Experimental results show that the HRL-GC architecture and MPC-PPO algorithm are suitable for large-scale air defense problems, effectively balances the effectiveness and dynamics of task assignment.

## Related work

### Deep reinforcement learning

Reinforcement learning was first introduced in the 1950s (Minsky, 1954) with the central idea of allowing an agent to learn in its environment and continuously refine its behavioral strategies through constant interaction with the environment and exploration by trial and error (Moos et al., 2022). With the continuous development of RL, algorithms such as Q-learning (Watkins and Dayan, 1992) and SARSA (Chen et al., 2008) have been proposed. However, when faced with problems in large-scale, high-dimensional decision-making environments, traditional RL methods also rapidly increase the computation, and storage space required to solve such problems.

Deep reinforcement learning is a combination of RL and deep learning (DL). DL enables reinforcement learning to be extended to previously intractable decision problems and has led to significant results in areas such as drone surveys (Zhang et al., 2022), recommender search systems (Shen et al., 2021), and natural language processing (Li et al., 2022), particularly in the area of continuous end-to-end control (Zhao J. et al., 2021). In the problem studied in this paper, the decisions shaped by the DRL for the agents must be temporally correlated, thus enabling the air defense task assignment strategy to maximize future gains and take the lead on the battlefield more easily.

### Hierarchical reinforcement learning

Hierarchical reinforcement learning (HRL) was proposed to solve the curse of dimensionality in reinforcement learning. The idea of this method is to decompose a whole task into multi-level subtasks by introducing mechanisms such as State space decomposition (Takahashi, 2001), State abstraction (Abel, 2019), and Temporal abstraction (Bacon and Precup, 2018) so that each subtask can be solved in a small-scale state space, thus speeding up the solution of the whole task. To model these abstract mechanisms, researchers introduced the semi-Markov Decision Process (SMDP) (Ascione and Cuomo, 2022) model to handle actions that must be completed at multiple time steps. The state space decomposition approach decomposes the state space into different subsets. It adopts a divide-and-conquer strategy for solving so that each solution is performed in a smaller subspace. Based on this idea, this paper divides the task assignment problem into two levels, scheduling and execution, and proposes the HRL-GC architecture to combine the advantages of centralized and distributed assignment effectively.

### Model-based reinforcement learning

Model-free RL does not require environmental models (e.g., state transfer probability models and reward function models) but is trained directly to obtain high-performance policies (Abouheaf et al., 2015). On the other hand, model-based RL is an approach that first learns the model during the learning process and then searches for an optimized policy based on that model knowledge (Zhao T. et al., 2021). Model-free RL is less computationally intensive at each iteration because it does not require learning model knowledge but has the disadvantage that too much invalid exploration leads to inefficient agents’ learning. Model-based RL methods can use a minimal number of samples to learn complex gaits, using the data collected to understand the model. The model is then used to generate a large amount of simulation data to learn a “state-action” value function to reduce the interaction between the system and the environment and improve sampling efficiency (Gu et al., 2016). For air defense scenarios, the sampling cost is high, and it isn’t easy to collect many data samples. Therefore, this paper uses a model-based RL approach to build a neural network model based on a small amount of sample data collected. The agent interacts with the model to obtain the data, thus reducing the sampling cost and improving the training efficiency.

### Model predictive control

Model predictive control (MPC) is a branch of optimal control (Liu S. et al., 2022), and the idea of MPC is widely used in model-based RL algorithms due to its efficiency in unconstrained planning problems. It is based on the specific idea of using the collected data to train a model and obtain an optimal sequence of actions by solving an unconstrained optimization problem (Yang and Lucia, 2021), as shown in Eq. 1.


(1) $$\begin{array}{c} a_{t}^{*},a_{t+1}^{*},\ldots ,a_{t+H}^{*}=\underset{a_{t},a_{t+1},\ldots ,a_{t+H}}{\arg\max}\sum_{k=0}^{H}r\,\left(s_{t+k},a_{t+k}\right)\\ s.t.s_{t+k+1}=\hat{f}\,\left(s_{t+k},a_{t+k}\right),k=0,1,\ldots ,H \end{array}$$

Where $\hat{f}\,\left(∙\right)$ is the learned model, the model is often a parametric neural network whose input is the current moment action *a*_*t*_, and the present moment state *s*_*t*_ outputs the predicted state ${\hat{s}}_{t+1}$ for the next moment; the loss function of the neural network can be constructed as (Yaqi, 2021)


(2) $$\varepsilon \,\left(\theta \right)=\frac{1}{\left|𝒟\right|}\,\sum_{\left(s_{t},a_{t},s_{t+1}\right)\in 𝒟}\frac{1}{2}\,{||\left(s_{t+1}-s_{t}\right)-{\hat{f}}_{\theta }\,\left(s_{t},a_{t}\right)||}^{2}$$

Where 𝒟 is the collected demonstration dataset, it is obtained by first generating random strategies to interact with the model, calculating the reward value for each policy, and selecting the sequence of actions with the highest cumulative reward. The first action of this sequence is then acted upon in the environment to obtain a new state, add the data to the demonstration dataset 𝒟, and repeat the same method to get the next action value. The model is trained using Eq. 2, and the dataset is continuously optimized, repeating the process until both the model and the taught dataset achieve good performance. By doing so, model errors and external disturbances can be effectively suppressed, and robustness can be improved (Nagabandi et al., 2018). Based on this idea, the MPC-PPO algorithm is proposed to train the model by the MPC method and then use the model to pre-train the network of PPO to improve the pre-training efficiency.

## Problem modeling

### Problem formulation

Modern large-scale air defense missions are no longer a one-to-one confrontation of one interceptor against one incoming target but rather a one-to-many and many-to-one confrontation accomplished through efficient organizational synergy in the form of tactical coordination. This is in response to saturated long-range attacks by cruise missiles and a multi-directional and multi-dimensional suppression attack by a mixture of human-crewed and uncrewed aircraft. However, this one-to-many and many-to-one confrontation assignment is not fixed; during air defense confrontations, the air attack offensive posture changes in real-time, and the confrontation assignment needs to be highly dynamic to respond to changes in the posture of the air attack threat (Rosier, 2009). The critical issue in this paper is the effective integration of combat resources according to the characteristics of different weapon systems and the ability to dynamically change the strategy according to the situation so that they can play a “1 + 1 > 2” combat effectiveness.

To reduce complexity while satisfying dynamism, this paper divides the air defense operations process into two parts, resource scheduling and mission execution, based on the idea of HRL. The complexity of the high-dimensional state-action space is reduced by decomposing the entire process into multiple more minor problems and then integrating the solutions to these problems into a solution to the overall task assignment problem.

### Markov Decision Process modeling of executive agents

In this paper, we study the air defense task assignment problem in a red-blue confrontation scenario, where the red side is the ground defender, and the blue side is the air attacker. We define a sensor and several interceptors around it as an interception unit. We use an independent learning framework to build the same MDP model for each interception unit.

State space: (1) states information of the defender’s defended objects; (2) resource assignment of the unit, sensor and interceptor states; (3) states information of the attacker’s targets within its own tracking and interception range; (4) states information of the attacker’s incoming targets that are assigned to it.

Action space: (1) what timing to choose to track the target; (2) which interceptor to choose to intercept the target; (3) how many resources to choose to intercept the target; and (4) what timing to choose to intercept.

Reward function: To balance the efficiency of exploration and learning of the agent, guiding the agent progressively toward the winning direction. This paper uses the principle of least resources to design the reward function.


(3) R=5⁢m+2⁢n-5⁢i+j

Where *m* is the number of human-crewed aircraft intercepted, *n* is the number of high threat targets blocked, *j* is the number of missiles intercepted, and *i* is the number of times our unit has been attacked as a result of a failed interception. Add five points for blocking staffed units, two points for intercepting high-threat targets, one point for intercepting missiles, and five points for each time our unit is attacked due to a failed interception.

### Markov Decision Process modeling of scheduling agents

The task of the scheduling agent is to coordinate the tracking and interception tasks to interception units based on the global situation, with a state space, action space, and reward function designed as follows:

State space: (1) states information of the defender’s defended objects; (2) states information of the defender’s interception units, including resource assignment, sensor and interceptor states, and states information of the attacker’s targets within the unit’s interception range; (3) states information of the attacker’s incoming targets; and (4) states information of the attacker’s units that can be attacked.

Action space: (1) select the target to be tracked; (2) select the target to be intercepted; (3) select the interception unit.

Reward function: The merit of the task assignment strategy depends on the final result of the task execution, so the reward of the scheduling agent is the sum of the tips of all the executing agents at the bottom plus the base reward, as shown in Eq. 4.


(4) $$R=\left\{ \begin{array}{c} \sum_{i=1}^{n}{\text{r}}_{i}\;\mathrm{Fail}\\ 50+\sum_{i=1}^{n}{\text{r}}_{i}\,\mathrm{Win} \end{array}\right.$$

Where _r_i__ is the bonus value earned by each executing agent, with a base bonus value of 50 points for a win and 0 points for a failure, the failure and victory conditions are described in Section “Experimental environment setting” based on the specific scenario. This paper uses a stage-by-stage approach of giving reward values to guide the agent to find the strategy that achieves victory. For example, the corresponding reward value is given after losing the blue side high-value unit. After the red side wins, it is given the winning reward value. This approach can increase the effect of maximizing global revenue on the agent’s revenue and reduce the agent’s self-interest as much as possible, enhancing robustness while ensuring the reliability of the strategy.

## Hierarchical architecture design for agents

### General structure

Reinforcement learning methods applied to task assignment can be broadly classified into two categories, centralized and distributed. The centralized idea is to extend the single-agent algorithm to learn the output of a joint action directly, but it is not easy to define how each of these agents should make decisions (Moradi, 2016). Distributed is where each agent learns its reward function independently, where for each agent, the other agents are part of the environment (Suttle et al., 2020). In large-scale air defense mission assignment problems, centralized methods can achieve globally optimal results but are often of low value for large-scale complex issues that are too costly in terms of time spent. Distributed algorithms, on the other hand, can negotiate a better result more quickly without having to have information about the specific parameters of individual weapons and the state of the surrounding environment. Still, they also face a significant problem: the assignment results are locally optimal and less globally coordinated for unexpected events (Wu et al., 2019).

**FIGURE 1:**
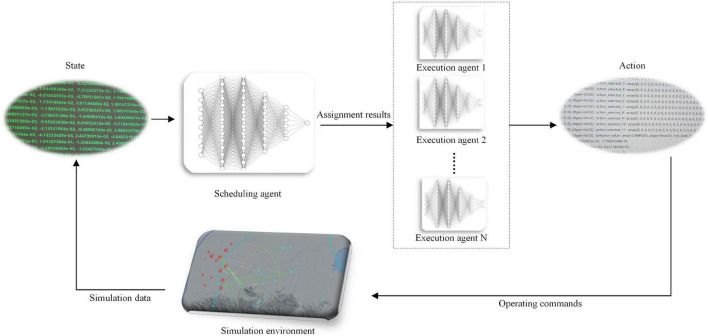
HRL-GC architecture.

To combine global coordination capability and high-speed computing capability, this paper follows the idea of OGMN architecture and proposes HRL-GC architecture. This architecture layers the agent into scheduling agents and executing agents, strengthening the autonomy of the underlying executing agent and making the assignment policy more reasonable, as shown in Figure 1.

The agent interacts with the environment to generate the simulation data, which the output port of the environment converts into state information as input to the scheduling agent; the high-level scheduling agent outputs the task assignment result and assigns the task to the underlying agent; the task execution agent outputs the final action information according to the assignment result and its state information; finally, the action information is then transformed into combat instructions according to the required data structure and input to the simulation environment. The above is a complete interaction process of the HRL-GC architecture at one time. We decompose the whole process into scheduling and execution, with different agents making decisions to reduce the complexity of the high-dimensional state-action space. The framework retain the global coordination capability of the centralized approach and add the efficient advantage of multiple agents. This method can preserve the scheduling agent’s coordination ability, avoiding missing key targets, duplicate shots, and wasted resources. Moreover, it can reduce the computational pressure of the scheduling agent and improve assignment efficiency.

### Design of a hierarchical training framework for agents

**FIGURE 2:**
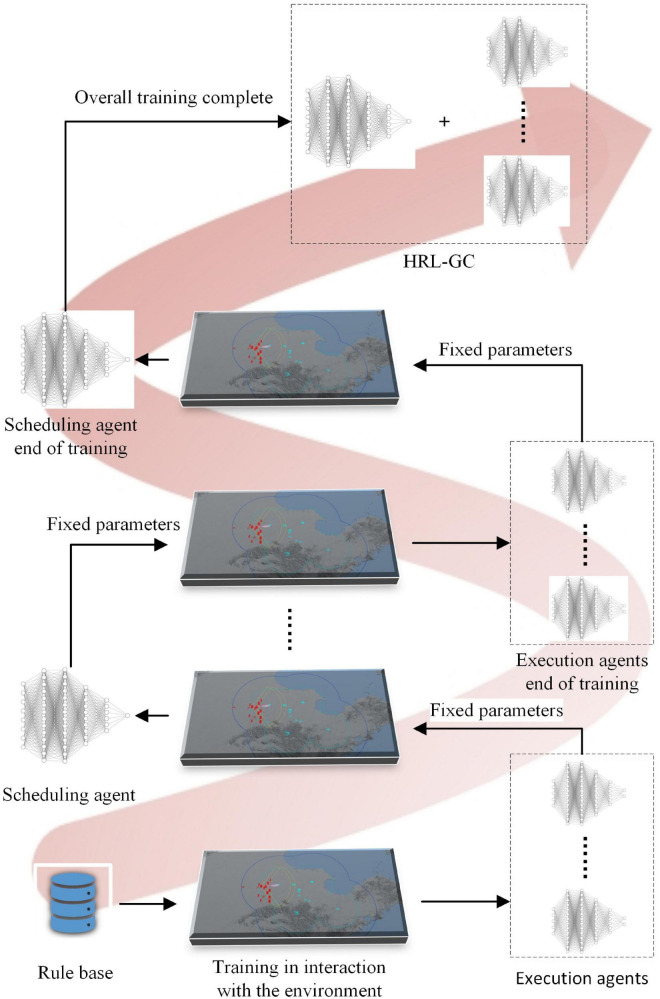
Hierarchical training framework.

Based on the idea of HRL, we need to train the scheduling and execution agents separately offline and then combine them for online inference. The reward function of the scheduling agent requires the reward values of all executing agents, which in turn depend to some extent on the outcome of the assignment of the scheduling agent. Therefore, the executive agent is trained to a certain level using expert assignment knowledge. All the executive agent’s network parameters are then fixed for introducing the scheduling agent, and the trained scheduling agent’s parameters are set for training the executive agent. The training framework is shown in Figure 2.

**FIGURE 3:**
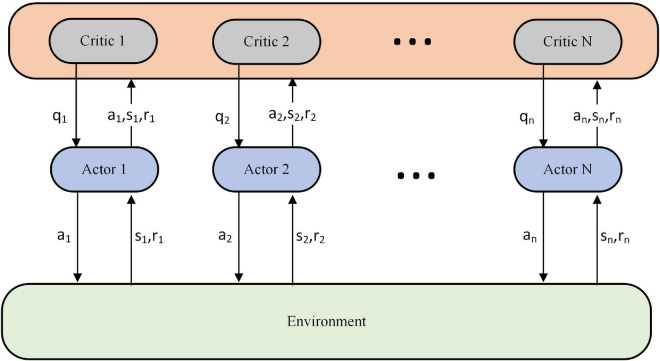
Centralized learning and decentralized execution.

The task assignment scheme of the knowledge rule base (Fu et al., 2020) is first used to train the underlying executing agent. When the executing agent reaches a certain level, the parameters of the executing agent are then fixed to train the scheduling agent. This paper is based on the Actor-Critic architecture (Fernandez-Gauna et al., 2022), which uses a centralized learning and decentralized execution approach for the training of multiple executing agents, as shown in Figure 3.

During training we use the *n*-value network (critic) to obtain actions, state observations and rewards for each executing agent, which are used to evaluate the decisions of the *n*-strategy network (actor). At the end of training, the critic is no longer used. The algorithm for executing the training of the agents is one of the critical issues studied in this paper and will be described in detail in Section “Model-based model predictive control with proximal policy optimization algorithm.” The training method for the scheduling agent refers to the proximal policy optimization for task assignment of general and narrow agents (PPO-TAGNA) algorithm in the literature (Liu J. Y. et al., 2022) to ensure the training effect and demonstrate more intuitively the changes the executive agent brings.

### Network architecture design for hierarchical reinforcement learning

**FIGURE 4:**
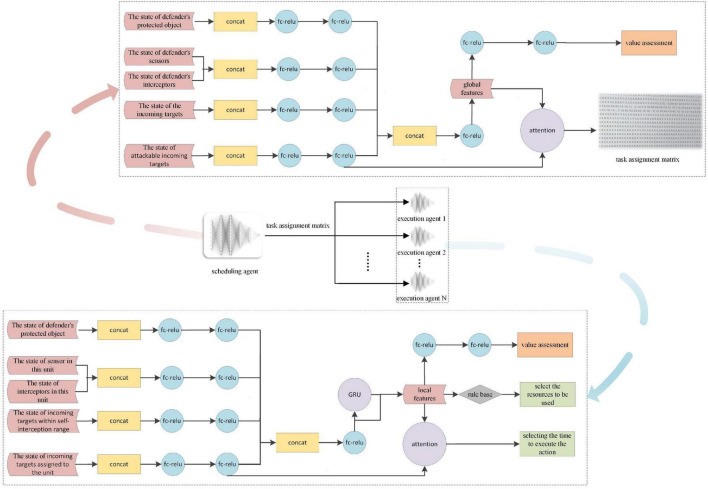
Network structure.

For DRL, the network structure of the agents is key to the research. For the HRL-GC architecture, we decoupled the general agent network in the OGMN into two parts. We improved them according to the MDP model in Sections “Markov Decision Process modeling of executive agents” and “Markov Decision Process modeling of scheduling agents” as the training networks for the scheduling and execution agents, respectively, as shown in Figure 4.

The input to the scheduling agents is global situational data, and the global features are obtained through feature extraction, vector concatenation, and other operations. After that, the global features output the value evaluation and task assignment results through two layers of FC-ReLU and attention mechanism operations, respectively. It is noteworthy that the actor’s output is changed from the real action to the task assignment matrix, i.e., the subject of the action, which significantly reduces the dimensionality of the action space and improves the computational speed.

The actor-network structure of the executing agent is the focus of this paper. Its input mainly consists of its state and the state of the assigned incoming target. After generating local features through feature extraction, vector connection, and Gated Recurrent Unit (GRU), it once again combines the information of the assigned target state to perform attention operations and finally outputs the timing of the execution of the task. We somewhat reuse the scheduling agent’s network and combine it vertically with a rule base, which is used to select the resources to be used. In this way, instead of using the rule base exclusively for decision making, we enhance the autonomy of the task execution agent, share more computational pressure on the scheduling agent, and make the assignment results more reasonable.

## Model-based model predictive control with proximal policy optimization algorithm

Sampling for large-scale adversarial tasks is a significant factor in excessive training time costs. The model-based RL approach can effectively improve this problem by building virtual models to interact with the agents. We use the MPC approach to train the virtual model, allowing the agent to interact with the model to obtain the demonstration data. To reduce the impact of model errors, we use only the demonstration data set to pre-train the network for the PPO algorithm, thus accelerating the exploration process during the initial training phase.

### Model predictive control approach for multi-agent task assignment

Based on the idea of MPC, this paper defines the entire task process time domain as [0,*nT*], and the system makes a decision every *T* moments. The time domain [0,*T*] is the period in which the task is executed. Before reaching the moment *T*, the agent needs to optimize the prediction of the strategy in the time domain [*T*,2*T*] based on the available situational information and resources. After reaching moment *T*, the agent executes the first action of the optimal action sequence while predicting and optimizing the decision solution for [2*T*,3*T*] in moment [*T*,2*T*], and so on until the end of the task.

Define the system as a synergy of m agents, *s*(*k*) denotes the states at moment *k*, μ(*k*) represents the command input in the period *k*,*k* + *T*,*f* is the resource selection model, and *k* = 0,*T*,2*T*…*nT* denotes the decision time point, then the discrete-time equation of state of the system is


(5) s⁢(k+T)=f⁢(s⁢(k),μ⁢(k)),s⁢(k)⁢μ⁢(k)∈∪

where the system state *s*(*k*) and the input decision μ(*k*) can be expressed as


(6) $$\left\{ \begin{array}{c} s\,\left(k\right)={\left[s_{1}\,\left(k\right),s_{2}\,\left(k\right),\cdots \,s_{m}\,\left(k\right)\right]}^{\text{T}}\\ \mu \,\left(k\right)={\left[\mu_{1}\,\left(k\right),\mu_{2}\,\left(k\right),\cdots \,\mu_{m}\,\left(k\right)\right]}^{T} \end{array}\right.$$

With *s*_*k+iT*_ denoting the predicted state of the control resource for the subsequent *iT* moments, using moment *k* as the current moment, the above equation shows that the expected state within [*k*,*k* + *T*] can be obtained based on the state *s*(*k*) at the moment *k* and the input decision μ(*k*), then Eq. 7 can be obtained.


(7) $$\begin{array}{c} s_{k+\left(i+1\right)\,T}=f\,\left(s_{k+i\,T},\mu_{k+i\,T}\right),\\ i=0,1\,\ldots \,H \end{array}$$

where *H* is the number of predicted steps.

Defining the value of the reward at moment *k* as r(*s*(*k*)), the global reward in the (*k*,*k* + *T*) time domain of *h* is


(8) $$\text{r}\,\left(s\,\left(k\right)\right)=\sum_{j=1}^{m}\text{r}\,\left(s\,\left(j|k\right)\right)$$

Where r(*s*(*j*|*k*)) is the reward of the jth agent at the moment *k*, this leads to an optimal task assignment model for the global system.


(9) $$\psi *\left(k\right)=\underset{\psi \,\left(k\right)}{\arg\max}\sum_{i=0}^{H}\text{r}\,\left(s_{k+i\,T},\mu_{k+i\,T}\right)\,\,s.t.$$

$$\left\{ \begin{array}{c} s_{k+\left(i+1\right)\,T}=f\left(s_{k+i\,T},\mu_{k+i\,T}\right),\left(i=0,1,\ldots ,H\right)\\ \psi \,\left(k\right)=\mu_{k},\mu_{k+T},\ldots \,\mu_{k+H\,T}\\ \psi^{*}\,\left(k\right)=\mu_{k}^{*},\mu_{k+T}^{*},\ldots \,\mu_{k+H\,T}^{*}\\ Y\,\left(s\,\left(k\right),\psi \,\left(k\right)\right)\leq 0 \end{array}\right.$$

Where *Y*(*s*(*k*),ψ(*k*))≤0 is the system constraint, which will be described in detail in Section “Constraints on the system.”

ψ*(*k*) is the action input sequence of the executing agent; the first action of this sequence, i.e., *μ*_k_*, is acted upon in the environment to obtain a new state. This round of data is added to the demonstration data set, and the next game of information is repeated using the MPC method, and so on until the end of the task. We then use the demonstration dataset 𝒟 to train model ${\hat{f}}_{\theta }$ *via* Eq. 10 and so on to continuously improve the quality of the taught dataset for the next step of network pre-training.


(10) $$\varepsilon \,\left(\theta \right)=\frac{1}{\left|𝒟\right|}\,\sum_{\left(s_{k},\mu_{k},s_{k+1}\right)\in 𝒟}\frac{1}{2}\,{||\left(s_{k+1}-s_{k}\right)-{\hat{f}}_{\theta }\,\left(s_{k},\mu_{k}\right)||}^{2}$$

### Model predictive control with proximal policy optimization algorithm

After training the model and obtaining samples using the MPC method, combined with the principles of the PPO algorithm, this method uses a pool of demonstration experience playback *R*_*d*_ to store this demonstration data and additionally constructs a pool of exploration experience playback *R*_*e*_ to store the exploration data of the agent. We obtained data from the two empirical playback pools mentioned above in a particular proportion. Considering the cumulative error of the model, the balance of demonstration data to the extracted data decreases with increasing time steps, and after 1,000 steps, the exploration data is used exclusively. The specific algorithm is described as shown in Algorithm 1.

**Algorithm 1:** Model predictive control with proximal policy optimization (MPC-PPO) algorithm.

Initialize the demonstration dataset *R*_*d*_, model ${\hat{f}}_{\theta }$ Repeat for *N* rounds Train ${\hat{f}}_{\theta }$ using data from *R*_*d*_ Repeat *T*-step Estimation of the optimal action sequence A using the MPC algorithm Interact the first action*a*_*t*_ in A with the environment to obtain the state*s*_*t* + 1_ Add (*s*_*t*_,*a*_*t*_,*r*_*t*_,*s*_*t* + 1_) to the data set *R*_*d*_ Initialize the policy parameters θ, *θ*_*old*_, and the exploration data pool *R*_*e*_ Repeat each round of updates Repeat for ε*N* Actors Repeat *t* steps Each step uses the old policy parameters *θ*_*old*_ to generate decisions The advantage estimate A is calculated in each step Store the sample data in _*R_e_*_ Iterate *K* steps Solving for the strategy gradient of the cumulative expected reward function Using small batches of data at a time, scaled from _*R_e_*_ and _*R_d_*_ The policy parameter _θ_ is updated with the policy gradient Update the new policy parameters to *θ*_*old*_

Where *θ*_*old*_ and θ refer to the old and new parameters, respectively, and in each iteration, the algorithm runs ε*N* Actors in parallel, with ε being the proportion of the total data explored. Each Actor runs *T* steps, collecting a total of ε*NT*. The dominance estimate *A*_1_…*A*_*T*_ is calculated at each step, and the remaining data is extracted from *R*_*d*_. After the data has been acquired, it will be used to update the policy parameters, iterating through each round and selecting small batches of data sets. Since, in the PPO algorithm, the data in the buffer needs to be emptied after x updates, a certain amount of demonstration samples need to be added after each emptying of the buffer. The proportion of demonstration samples decreases as the number of updates increases so that the impact of the cumulative error of the model can be reduced to a certain extent.

### Constraints on the system

Air defense tasks require the highest safety level in policy and maximum avoidance of unsafe maneuvers during training. Therefore, to suppress the uncertainty in the model learning process and make the model error smaller, we also need to add some constraints to the system to satisfy the realism and safety of the model. The specific rules are as follows:

(1) Cooperative guidance constraints

For multi-platform cooperative systems, the constraints on unified guidance accuracy and distance must be satisfied during suitable guidance, as shown in Eq. 11.


(11) $$\left\{ \begin{array}{c} \theta_{T}\in \theta_{g\,u\,i\,d\,e},T\in \left\{1,2,\ldots ,n\right\}\\ \sigma_{T}\geq \sigma_{\min},T\in \left\{1,2,\ldots ,n\right\}\\ {⋃}_{i=m}S_{i}\geq S_{T},T\in \left\{1,2,\ldots ,n\right\} \end{array}\right.$$

Where *n* denotes the number of missiles to be guided, *θ*_*T*_ represents the set of flight airspace angles of the target missile, *θ*_*guide*_ denotes the operating range of the sensor, σ_*T*_ denotes the guidance accuracy, σ_*min*_ denotes the minimum guidance accuracy requirement, *S*_*i*_ means the guidance distance of the sensor, and *S*_*T*_ represents the distance of the missile. That is, the constraints of minimum guidance accuracy and maximum guidance distance must be satisfied during cooperative guidance.

(2) Time constraints

Due to the highly real-time nature of air defense tasks, task assignment is highly time-constrained, and, for the executing agent, the factors associated with the time constraint are mainly reflected in

1. Timing of interceptions

Longest interception distance:


(12) $$D_{L\,I}=\sqrt{D_{L\,S}^{2}+{\left(v_{m}\,t_{L}\right)}^{2}+2\,v_{m}\,t_{L}\,\sqrt{D_{L\,S}^{2}-\left(H^{2}+P^{2}\right)}}$$

Nearest interception distance:


(13) $$D_{N\,I}=\sqrt{D_{N\,S}^{2}+{\left(v_{m}\,t_{N}\right)}^{2}+2\,v_{m}\,t_{N}\,\sqrt{D_{N\,S}^{2}-\left(H^{2}+P^{2}\right)}}$$

Where *v*_*m*_ is the speed of the target, *H* is the altitude of the target, *P* is the shortcut of the target’s flight path, *D*_*LS*_ and *D*_*NS*_ are the target’s kill zone oncoming far boundary and the target’s kill zone oncoming near the border, and *t*_*L*_ and *t*_*N*_ are the times the target flies to the distant and near edges of the oncoming kill zone, respectively.

2. Timing of sensor switch-on:

Sensor detection of the target is a prerequisite for intercepting the target. In combat, it takes a certain amount of time, called pre-interception preparation time *t*_*P*_, from sensor detection to interceptor’s interception of the target.

The required distance for sensors to find a target *D*_*S*_ is based on the length of the target at the furthest encounter point.


(14) $$D_{S}=\sqrt{D_{N\,S}^{2}+v_{m}^{2}\,{\left(t_{L}+t_{P}\right)}^{2}+2\,v_{m}\,\left(t_{L}+t_{P}\right)\,\sqrt{D_{N\,S}^{2}-\left(H^{2}+P^{2}\right)}}$$

We define the state that satisfies the security constraint as *S*, which gives us Eq. 15.


(15) $$\left\{ \begin{array}{c} Y\,\left(s\,\left(k\right),\psi \,\left(k\right)\right)>0\;s_{t}⊄S\\ Y\,\left(s\,\left(k\right),\psi \,\left(k\right)\right)\leq 0\;s_{t}\subset S \end{array}\right.$$

In this multi-platform collaborative system, assignments can only be made when *Y*(*s*(*k*),ψ(*k*))≤0; otherwise, assignments against this batch of targets are invalid.

## Experiments and results

### Experimental environment setting

As an example of a large-scale air defense mission, the red side is the defender, with seven long-range interception units and five short-range interception units to defend a command post and an airfield. The long-range interception unit consists of one long-range sensor and eight long-range interceptors, and the short-range interception unit consists of one short-range sensor and three short-range interceptors. Blue is the attacker, setting up 18 cruise bombs, 20 UAVs, 12 fighters, and 2 jammers to attack Red in batches. Red loses when Red’s command post is attacked three times; Red loses when the distance between Blue bombers and Red’s command post is less than 10 km; Red loses when Red’s sensor losses exceed 60%; Red wins when Blue loses more than 30% of its fighters.

**FIGURE 5:**
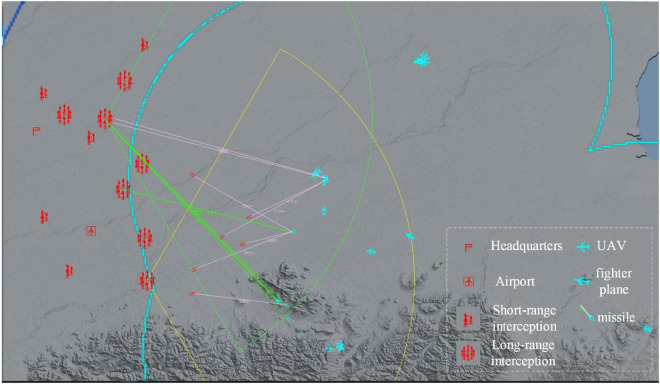
Schematic diagram of an experimental scenario.

A schematic diagram of the experimental scenario is shown in Figure 5.

In the experiments in this paper, the agent’s interaction with the environment takes place on the digital battlefield. The digital battlefield is a DRL-oriented air defense combat simulation framework, which is responsible for the presentation of the battlefield environment and the simulation of the interaction process, including the simulation of the behavioral logic of each unit and the damage settlement of mutual attacks. It supports operations such as combat scenario editing and configuration of weapon and equipment capability indicators, allowing agents to be trained in different random scenarios. And physical constraints such as earth curvature/obscuration can be randomly changed within a certain range.

### Experimental hardware configuration

The CPU running the simulation environment is an Intel Xeon E5-2678v3, 88 core, 256 G memory; GPU × 2, model Nvidia GeForce 2080Ti, 72 cores, 11G video memory. In PPO, the hyperparameters is ε = 0.2, the learning rate is 10^–4^, the batch size is 5,120, and the number of hidden layer units in the neural network is 128 and 256.

### Agent architecture comparison

**FIGURE 6:**
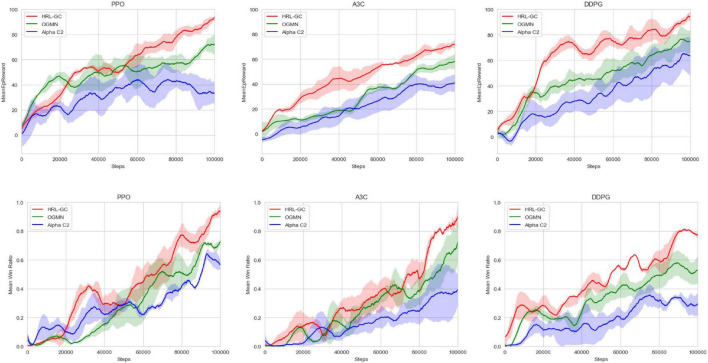
Comparison of agent architecture training effect.

Alpha C2 (Fu et al., 2020) uses a commander structure, to which OGMN (Liu J. Y. et al., 2022) adds rule-driven the narrow agent, and the HRL-GC architecture proposed in this paper uses data-driven the narrow agent on top of OGMN. Therefore, in this experiment, we first trained the execution agents 50,000 times using a rule base and fixed parameters and then used three different algorithms to verify the differences in training efficiency between the three agent architectures. We iterated the three architectures 100,000 times on the digital battlefield using the PPO, A3C, and DDPG algorithms, respectively, collecting data from each game of the confrontation and counting the reward values and win rates obtained by the red-side agents. The results are shown in Figure 6.

Comparing the mean reward curves shows that the HRL-GC architecture can significantly improve the training efficiency, and the final mean reward value is higher. The reward curve is more likely to stabilize. In terms of win ratio, the proposed agent architecture also achieves higher win ratios faster and is more likely to stabilize than Alpha C2 and OGMN. Experiments have demonstrated that the HRL-GC architecture further improves training efficiency and agents’ decision-making while retaining the ability to coordinate.

### Algorithm performance comparison

#### Comparison of training data

**FIGURE 7:**
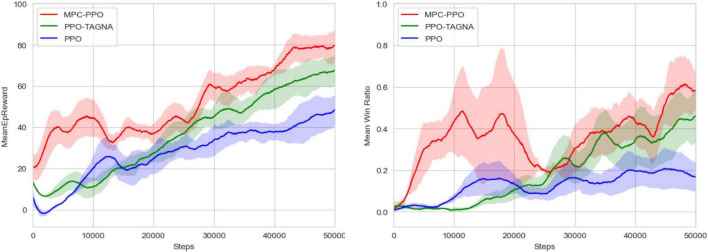
Algorithm performance comparison.

To verify that the MPC-PPO algorithm proposed in this paper can improve the efficiency of the pre-training period, we first trained the scheduling agent 50,000 times using the PPO-TAGNA algorithm based on the HRL-GC architecture in the same scenario setting. Then, the execution agent performed the MPC-PPO algorithm, the PPO-TAGNA algorithm, and the PPO (Fu et al., 2020) algorithm 50,000 times for centralized training. The training results are shown in Figure 7.

The comparison results show that the MPC-PPO algorithm proposed in this paper can achieve a higher initial reward and a significant increase in win ratio in the early stages. Both the reward value curve and the win ratio curve of the training have an inevitable decline and are not very stable after the rise due to model errors and other factors; however, in general, MPC-PPO is more efficient in the first 50,000 steps of training compared to PPO-TAGNA and PPO and can achieve a faster increase in the reward value and win ratio obtained by the agent.

#### Behavioral analysis

**FIGURE 8:**
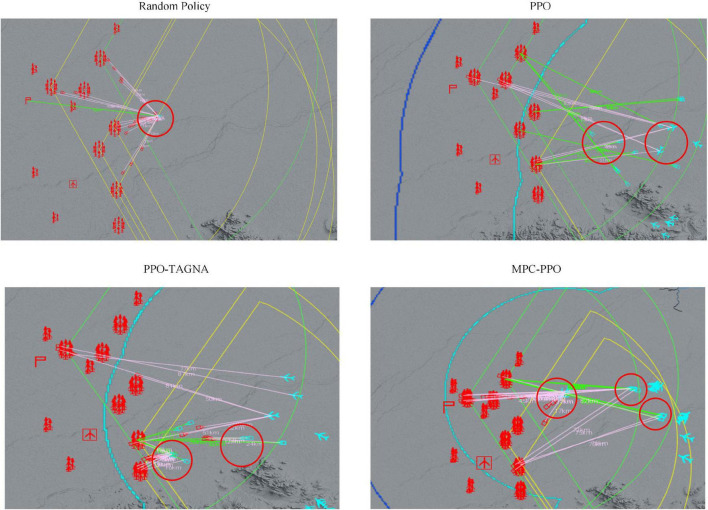
Comparison of behavioral details.

The model-based RL approach aims to allow the agent to reduce ineffective exploration in the initial training stages and reach a certain level quickly. So we trained only the PPO agent, the PPO-TAGNA agent, and the MPC-PPO agent proposed in this paper 50,000 times in a complex scenario. We then performed behavioral analysis separately and compared them with the untrained agent. The results are shown in Figure 8.

The behavioral analysis shows that the untrained agents (top left) adopt a random policy, wasting too much ammunition in defending against the first attacks and eventually failing because they run out of resources; the PPO agents (top right) have not yet explored a mature policy at this stage and only attack high-value targets without dealing with incoming high-threat targets; the PPO-TAGNA agents (bottom left) at this stage The MPC-PPO agents (bottom right) has learned the strategy of coordinated interception, but the response timing is inaccurate, and the scope of coordination is small; the MPC-PPO agents (bottom right) at this stage can effectively coordinate the interception of high-threat targets while attacking high-value targets. Therefore, the MPC-PPO algorithm in large-scale complex scenarios enables the agents to reduce ineffective exploration in the initial stages of training and learn practical policies more quickly.

## Conclusion

To address the problem that modern air defense task assignment is difficult to balance effectiveness and dynamism, this paper proposes the HRL-GC architecture, which layers the agents into a scheduling agent and execution agents, with the scheduling agent coordinating the global situation to ensure effectiveness and the execution agent distributing the execution to improve efficiency and thus ensure dynamism. To enhance the efficiency of the initial stage of agents training, this paper proposes a model-based MPC-PPO algorithm to train the execution agents. Finally, experiments compare the agent framework and the algorithm’s performance in a large-scale air defense scenario. The experimental results show that the HRL-GC architecture and MPC-PPO algorithm can further improve the decision-making level of the agents and train them more efficiently. The assignment scheme is more in line with the needs of large-scale air defense, effectively balancing the effectiveness and dynamics of air defense task assignment.

## Data availability statement

The raw data supporting the conclusions of this article will be made available by the authors, without undue reservation.

## Author contributions

J-yL: conceptualization, software, and writing—original draft preparation. J-yL and GW: methodology. J-yL, QF, and X-kG: validation. S-yW: formal analysis. X-kG: investigation. QF: resources, project administration, and funding acquisition. J-yL and S-yW: data curation and visualization. GW and QF: writing—review and editing. GW: supervision. All authors read and agreed to the published version of the manuscript.
